# Synthesis, Structural Investigations, and In Vitro/In Silico Bioactivities of Flavonoid Substituted Biguanide: A Novel Schiff Base and Its Diorganotin (IV) Complexes

**DOI:** 10.3390/molecules27248874

**Published:** 2022-12-14

**Authors:** Zahoor Abbas, Manoj Kumar, Hardeep Singh Tuli, Essam M. Janahi, Shafiul Haque, Steve Harakeh, Kuldeep Dhama, Pallvi Aggarwal, Mehmet Varol, Anita Rani, Shashi Sharma

**Affiliations:** 1 Department of Chemistry, Maharishi Markandeshwar University, Sadopur, Ambala 134007, India; 2 Department of Biotechnology, Maharishi Markandeshwar Engineering College, Maharishi Markandeshwar (Deemed to Be) University, Mullana, Ambala 133207, India; 3 Independent Researcher, Building 1848, Road 7542, Al Janabiyah 0575, Bahrain; 4 Research and Scientific Studies Unit, College of Nursing and Allied Health Sciences, Jazan University, Jazan 45142, Saudi Arabia; 5 King Fahd Medical Research Center, Yousef Abdullatif Jameel Chair of Prophetic Medicine Application, Faculty of Medicine, King Abdulaziz University, Jeddah 21589, Saudi Arabia; 6 Division of Pathology, ICAR-Indian Veterinary Research Institute, Bareilly 243122, India; 7 Department of Chemistry, Pt. CLS Govt. College, Karnal 132001, India; 8 Department of Molecular Biology and Genetics, Faculty of Science, Kotekli Campus, Mugla Sitki Kocman University, Mugla TR48000, Turkey; 9 Department of Chemistry, IEC University, Baddi 174103, India; 10 Department of Chemistry, Dyal Singh College, Karnal 132001, India

**Keywords:** Schiff base, diorganotin derivatives, biological activities, molecular docking

## Abstract

Quercetin is one of the most powerful bioactive dietary flavonoids. The in vivo biological study of quercetin is extremely difficult due to its very low solubility. However, diorganotin complexes of quercetin are more useful when contrasted with quercetin due to increased solubility. In the present study, quercetin, substituted biguanide synthesized in the form of Schiff base and its di-alkyl/aryl tin (IV) complexes were obtained by condensing Schiff base with respective di-alkyl/aryl tin (IV) dichloride. Advanced analytical techniques were used for structural elucidation. The results of biological screening against Gram-positive/Gram-negative bacteria and fungi showed that these diorganotin (IV) derivatives act as potent antimicrobial agents. The in silico investigation with dihydropteroate (DHPS) disclosed a large ligand–receptor interaction and revealed a strong relationship between the natural exercises and computational molecular docking results.

## 1. Introduction

**Figure 1:**
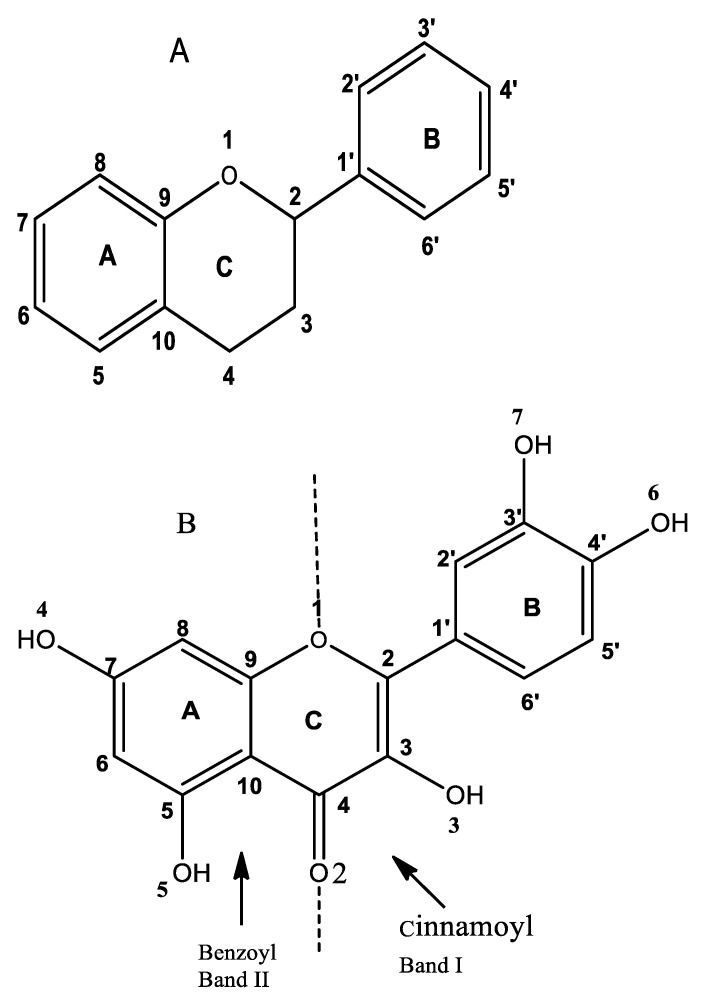
(**A**) General structure of flavonoids. (**B**) Chemical structure of quercetin; divisions of bands I and II; Ring A (Band II) benzoyl system; Ring B (Band I) cinnamoyl system.

Flavonoids are polyphenolic compounds found in many plants, consisting of a three-carbon chain framework connected together by two phenyl rings (C_6_-C_3_-C_6_); two C_6_ groups are two benzene rings containing a pyran ring (Figure 1A). Flavonoids are categorized into flavonols, flavanones, flavones, isoflavones, chalcones, catechins, and anthocyanidins on the basis of chemical structure. Flavonoids are associated with a wide range of promising impacts and found to be a basic component in a variety of nutraceutical, drug, and restorative applications. The dietary intake of flavonoids may result in a reduced risk of different types of cancers, such as gastric, breast, prostate, and colorectal cancer. In addition, they are known to be powerful inhibitors for a few proteins, such as xanthine oxidase (XO), cyclooxygenase (COX), lipoxygenase, and phosphoinositide 3-kinase. Currently, as cellular dysfunction is linked to cell proliferation in an oxidative environment, it is hypothesized that compounds that can prevent the actions of ROS-producing enzymes, such as xanthine oxidase and lipoxygenase, are significant in the development of new drugs [1,2,3,4].

One of these flavones is quercetin (3,3′,4′,5,7-pentahydroxyflavone) (Figure 1B). Quercetin has been found to act as a very significant chemotherapy agent with some tremendous pharmacological activities, such as anti-cancer [5,6], antimicrobial, and antitumor [7,8]. Moreover, quercetin is one of the compelling metal chelators with three potential chelating positions, i.e., 3 & 4, 5 & 7, and 3′ & 4′. Furthermore, it has been observed that complexing of quercetin with metal upgrades its pharmacokinetic reactions both in vitro and in vivo [9,10,11,12,13,14].

Although quercetin has a powerful pharmacological potential, its low solubility makes it difficult to act as biocidal agent [15,16]. Consequently, few investigations have been performed to overcome the problem [17,18,19,20,21]. During the last several years, quercetin Schiff base metal complexes have been broadly investigated and found to have great biological potential [22,23,24,25,26].

On the other hand, organotin complexes are the most diverse segment of chemical compounds with applications ranging from material science and catalytic activities to therapeutic agents for various diseases due to their wide variety of interesting structural possibilities [27]. Organotin compounds occupied an important place in cancer chemotherapy reports [28] due to their cytotoxic effects, ability to bind with DNA, anti-proliferating nature, and apoptotic-inducing nature. Organotin complexes, especially with Schiff base ligands, have been screened for their role in anti-microbial and anti-inflammatory activities [29].

The chemical structures of various reported organometallic complexes of flavonoids support the magnificent radical scavenging ability. Valentina Uivarosi et al. found that the metal-flavonoid complexes have enhanced the physiochemical and biological properties of flavonoids [30]. In another study, Andrea Kurzwernhart et al. reported that the organometallic complexes of flavanol scaffold employing O,O chelating ligand system seizes the in vitro anticancer activity [31]. Considering the biological importance of these compounds and the challenges due to the poor solubility of quercetin, the present study discloses the synthesis, spectral investigations, biological screening, and in silico study of diorganotin (IV) derivatives of novel Schiff base, where Schiff base is obtained from quercetin and biguanide. The results of the in silico study strongly support the potential of synthesized complexes as biocidal agent [32].

## 2. Results and Discussion

### 2.1. Infrared Spectra

The coordination of Schiff base ligand (H_2_L) with the Sn atom was verified by evaluating the IR spectra of ligand and complexes (**1**–**4**) (Figure S2). The IR spectra of ligand displaced a broad band at 3394–3370 cm^−1^ confirming v(O-H) stretching vibration of coordinated water molecules and -OH groups of the ligand. The absorption band obtained in Schiff base ligand (H_2_L) at 1657 cm^−1^ was found to be absorbed at lower values by 20–30 cm^−1^ and observed at 1627–1637 cm^−1^ in the spectra of diorganotin (IV) complexes [33,34]. The band at 1299 cm^−1^ in spectra of Schiff base ligand was attributed to v(C-O-C) stretching which remains unchanged in the spectra of diorganotin complex; therefore, indicating that there is no involvement of some oxygen atom of any ring in complexation. Furthermore, the bands obtained at values 501–520 cm^−1^ and 460–480 cm^−1^ were attributed to v(Sn-O) and v(Sn-N) vibrations, respectively [35,36] and, indeed, confirm the co-ordination between tin and Schiff base ligand.

### 2.2. HNMR Spectra

The ^1^HNMR spectra of complexes recorded in DMSO-d6 are found to exhibit peaks according to the proposed structure(Figure S1). The absence of peak for the free 3-OH indicates deprotonation of the hydroxyl groups due to bonding with Sn (IV). The chemical shifts at 12.18–12.21 and 8.17–8.21 ppm proved that 5-OH and 7-OH groups are still present in the complexes (**1**–**4**) [37]. The aromatic protons of H_2_L and derivatives seemed identical and were observed as multiplets in the range of 6–7 ppm, indicating non-participation of aromatic ring in complex formation. The spectra of complexes consisted of new shifts at 0.79, 0.90, 0.98, and 7.36 ppm, and were attributed to -CH_3_, -C_2_H_5_, -C_4_H_9_, and phenyl protons, respectively [38].

### 2.3. C NMR Spectra

The ^13^C NMR spectra confirmed the resonance of aromatic rings in the range of 115–157 ppm for the ligand and remained undisturbed in the spectra of complexes reflecting the non-participation of aromatic ring in bond formation with the central Sn atom. The height at 148.9 ppm, which was attributed to 3-OH carbon in the spectra of ligand, received a limit in the spectra of complexes suggesting its bonding with the tin atom. The spectra of complexes consisted of new signals due to carbon-tin bonding, at 8.51 due to -CH_3_ carbon, 7.5, 33.1, and 147.9–124.1 ppm for -C_2_H_5_, -C_4_H_9_, and phenylcarbon atoms, respectively [34].

### 2.4. Sn NMR Spectra

The chemical shift value of ^119^Sn NMR describes the coordination variety of complexes. Signal for Sn was determined at δ −126.03 in the spectra of methyl complexes, whereas at δ –156.04 ppm in ethyl and at δ −290.25 and −310.04 ppm in the complexes of butyl and phenyl, respectively [39]. These values of chemical shift certainly prove that the Penta-coordinated surroundings for tin atom, and additionally all the indicators appear as singlet, which confirms the existence of one isomer of mononuclear complexes of tin (IV) metal [40,41,42,43].

### 2.5. Mass Spectra

LCMS technique has been utilized for the determination of mass, and thus the molecular weight of the compounds. The spectra of the ligand contained a peak at m/z = 669.57, which has been attributed to [M + H] ion accompanied by the nitrogen rule and, currently, in full settlement with the proposed molecular mass. Mass spectrometry of the complexes showed the formation of [Me_2_SnL], [Et_2_SnL], [Bu_2_SnL], and [Ph_2_SnL] through displaying peaks at m/z = 819.05, 845.49, 901.49, and 941.46.

### 2.6. UV-Visible Spectroscopic Study of the Complexes

The UV-visible spectrum of H_2_L indicated two primary absorption bands at 365 nm (band I) and 262 nm (band II). Band I is associated with ring B (cinnamoyl system) and band II with ring A (benzoyl system), Figure 1 [44,45,46]. The depth of H_2_L (band I) decreases progressively with the addition of tin (IV) derivatives, and new absorption peaks appeared at 410, 414, 420, and 430 for methyl, ethyl, butyl, and phenyl, respectively. These shifted peaks indicated the coordination of tin (IV) to the 3-hydroxyl groups of ring C [47]. Band I bathochromic shift can be defined by the interaction of tin (IV) with the 3-OH involving ring C, producing electrical redistribution between H_2_L and its derivatives, which resulted in the π-bonding. The 5-OH does not involve (i) 3-OH with higher chelation potential than the 5-OH and (ii) the delocalization of the oxygen electrons of the 3-OH involvement was greater than 5-OH [48].

### 2.7. Antimicrobial Assay

In vitro antimicrobial screening tests of the synthesized ligand and its diorganotin (IV) derivatives have been carried out against four bacterial strains; two Gram-positive (*Staphylococcus aureus*, *Bacillus subtillis*) and two Gram-negative (*Escherichia coli, Pseudomonas aeruginosa*), as well as two fungal species (*Scopulariopsis canadensis*, *Aspergillus niger*). All tests were carried out in triplicates. Measurements of the inhibition zones were recorded as a mean value of three readings, which are shown in Table 1. Based on the pharmacological data, it has been observed that complex three or four had positive antimicrobial activities against all the examined species with better efficacy than the antibiotics used as standard (ciprofloxacin and fluconazole). Due to the presence of azomethine linkage (>C=N-), complexes have been found to inhibit microbial growth. Different mechanisms have been suggested including the inhibition of cell wall synthesis and loss of the bioactivity of fundamental enzymes, such as dihydropteroate synthase [49,50]. In the current study, the examined ligand and their metal complexes possess strong anti-microbial activities. Promising antimicrobial activities of the metal complexes may be due to the chelation ability of the ligand [51]. As per the theory, chelation effects result in enhancement of permeability of the drug into the cytoplasm of the cell [52]. Bu_2_SnL and Ph_2_SnL derivatives have a promising antimicrobial action in contrast to ligand alone. The results indicated that phenyl by-product (**4**) acts as an enhanced anti-microbial agent with maximum zone of inhibition, i.e., 30 mm with MIC value of 79 ppm in evaluation to butyl by-product (29 mm and 84 ppm). This effect may be due to the delocalization of π-electrons and lipid soluble nature of tin atoms, which facilitate their entry into the microbial cells [53,54]. Therefore, upon evaluating the antimicrobial ability of the diorganotin (IV) complexes and conventional antibiotics (ciprofloxacin and fluconazole), it was found that complexes (**3** & **4**) have considerable activity against the tested microbes, in contrast to the antibiotics that demonstrated their use as effective antibacterial/antifungal agents.

### 2.8. Molecular Docking Studies

**Figure 2:**
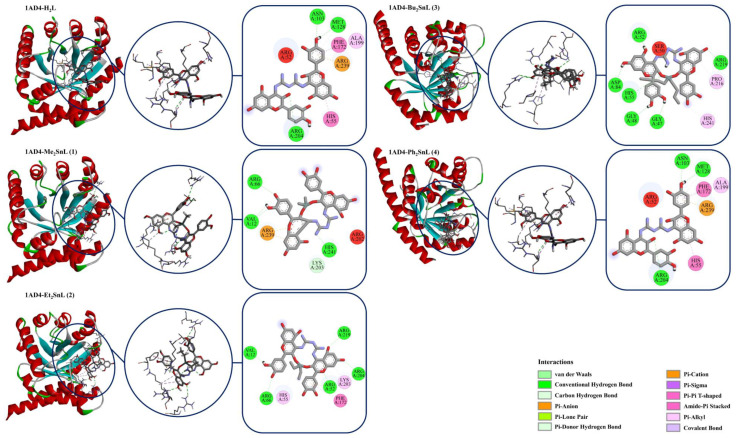
Molecular docking results of the complexes with the enzyme *Dihydropteroate synthase* (DHPS) from *Staphylococcus aureus* (PDB ID: 1AD4).

**Figure 3:**
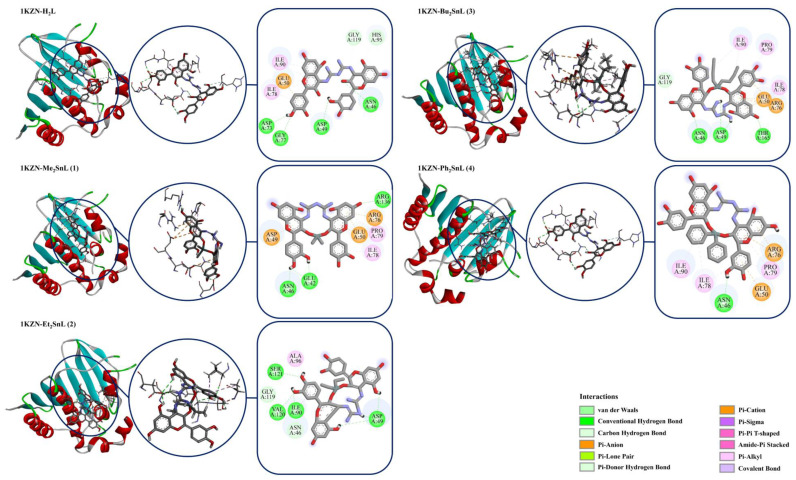
Molecular docking results of the complexes with *Escherichia coli* 24 kDa DNA gyrase domain (PDB ID: 1KZN).

*Staphylococcus aureus* is widely known as a Gram-positive bacterium that is a frequent cause of life-threatening infections in hospitals, such as bacteremia, toxic shock syndrome, infective endocarditis, osteomyelitis, gastroenteritis, meningitis, septic arthritis, respiratory tract infections, pulmonary infections, skin and soft tissue infections, urinary tract infections, and prosthetic device infections [55,56]. Therefore, dihydropteroate synthase (DHPS) was targeted for the molecular docking studies. The enzyme DHPS performs a key function in microbial folate biosynthesis through the manufacturing of 7,8-dihydropteroate from dihydropterin pyrophosphate (DHPP) and para-aminobenzoic acid (pABA) [57]. The loss of the bioactivity of DHPS is suggested as one of the main mechanisms of the inhibition of microbial growth [46,47]. The X-ray crystal structure of the dihydropteroate synthase (DHPS) from staphylococcus aureus (1AD4) was used as a possible target for the synthetized Schiff base and its diorganotin (IV) complexes [58,59,60,61,62]. Although the synthesized ligand (H_2_L) displayed the strongest binding affinity (−9.7 kcal/mol) to the DHPS (PDB ID: 1AD4), the in vitro antimicrobial activity of H_2_L was determined as lower than the synthesized complexes. Consistent with the literature, quercetin’s low solubility appears to suppress its extensive biocidal action [15,16]. Moreover, the synthesized Me2SnL (**1**) and Bu2SnL (**3**) showed the weakest binding properties with binding affinities of 8.4 kcal/mol (Figure 2 and Figure 3 and Table 2), although Bu2SnL (**3**) was determined as one of the most effective antimicrobial agent on *S.aureus* with maximum zone of inhibition, i.e., 29 mm with MIC value of 84 ppm (Table 1). Therefore, it is hypothesized that Bu2SnL (**3**) triggers additional antimicrobial mechanisms to enhance its biocidal capacity, and should be further investigated. On the other hand, the synthesized Ph2SnL (**4**) was determined as the most effective complex among the synthesized complexes with binding affinity of −8.9 kcal/mol in correlation with in vitro experimental results (Table 1 and Table 2; Figure 2).

DNA gyrase subunit B (1KZN) is considered to be a very important target for antibacterial drug design and discovery [63]. DNA gyrase is an important bacterial protein that is involved in the replication, transcription, and the catalysis of the negative supercoiling of bacterial circular DNA [64]. Therefore, the crystal structure of *E. coli* 24 kDa DNA gyrase fragment was used as a possible target for the synthetized Schiff base and its diorganotin (IV) complexes. Similar to the docking studies of 1AD4, the synthesized ligand (H_2_L) showed the strongest binding affinity (−9.5 kcal/mol) to the DNA gyrase subunit B, although the in vitro antimicrobial activity of H_2_L on *E. coli* was lower than the synthesized complexes (Table 1 and Table 2). Additionally, it was determined that the data obtained in molecular docking studies performed with DNA gyrase (1KZN) were not compatible with the results of antimicrobial tests on *E. coli*. Although molecular docking studies indicated that Et_2_SnL (−9.0 kcal/mol) and Bu_2_SnL (−8.5 kcal/mol) are the most active ones among the synthesized complexes, respectively, the antimicrobial tests showed that Ph_2_SnL (**4**) and Bu_2_SnL (**3**) had respectively more antimicrobial activity on *E. coli* than the others (Table 1 and Table 2; Figure 3). It seems that the antimicrobial activity of the synthesized complexes is not directly related to the effect on DNA gyrase enzyme.

**Figure 4:**
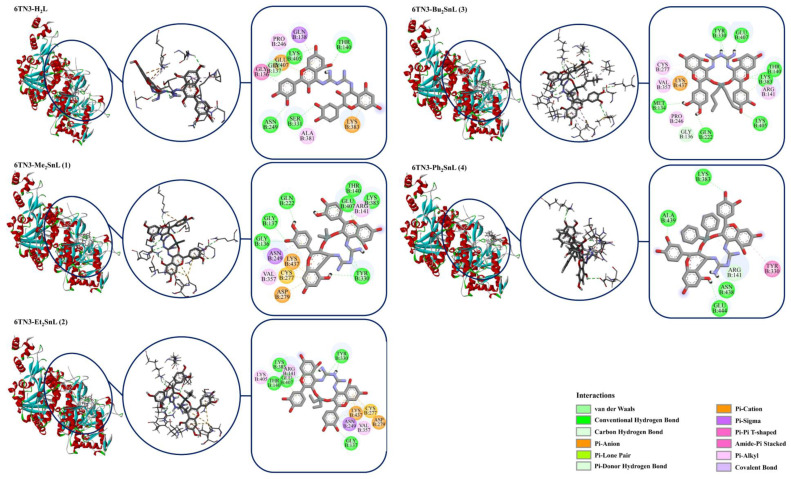
Molecular docking results of the complexes with the *Aspergillus fumigatus* UDP-*N-*acetylglucosamine pyrophosphorylase (PDB ID: 6TN3).

Aspergillosis is known as an invasive and potentially life-threatening infection caused by *Aspergillus*, and frequently observed in immunocompromised patients [65]. UDP-*N*-acetylglucosamine pyrophosphorylase (UAP1) was reported as a potential drug target for *Aspergillus fumigatus* since it plays substantial roles in the biosynthesis of fungal cell wall by catalyzing the biosynthesis of uridine diphosphate-N-acetylglucosamine (UDP-GlcNAc), converting UTP and GlcNAc-1P to the sugar nucleotide [66]. Therefore, the crystal structure of UDP-N-acetylglucosamine pyrophosphorylase (6TN3) from *Aspergillus fumigatus* was used as a possible target for the synthetized Schiff base and its diorganotin (IV) complexes. The molecular docking studies showed that Me_2_SnL, Et_2_SnL, and Bu_2_SnL have similar binding affinities to the UAP1 by −9.2, −9.2, and −9.1 kcal/mol, respectively (Table 2 and Figure 4). Although the synthesized complexes show strong binding affinities on UAP1, their in vitro antifungal activities are not very compatible with the molecular docking results (Table 1 and Table 2). For example, Ph_2_SnL was determined as the most effective antifungal complex among the synthesized diorganotin (IV) complexes, although its binding affinity was determined as the weakest one (−7.3 kcal/mol). Consequently, it is clear that there is a need to further investigate alternative drug targets in order to fully understand the antimicrobial activity mechanisms of the synthesized complexes, and it should be noted that there are complicated biological and chemical processes for the emergence of antimicrobial activities of drugs.

## 3. Experimental

### 3.1. Materials and Measurements

Diorganotin dichloride and quercetin.2H_2_O were purchased from Sigma Aldrich (USA). Pure biguanide was prepared from biguanide sulphate. All other solvents have been purchased from Merck (INDIA) and dried before use, according to the preferred strategies [67]. The spectroscopic evaluation was performed at SAIF lab Panjab University, Chandigarh. Elemental analysis was carried out using Perkin-Elmer instrument and IR spectral investigations were achieved with KBr pellet. The NMR (^1^H, ^13^C, ^119^Sn) study was carried out using Bruker Avance 500 MHz spectrometer via dimethyl sulfoxide (DMSO) as solvent and tetramethylsilane (TMS) as internal standard. Melting points have been determined in open capillary tubes. The mass spectrometry was determined on the TESQ-8000 mass spectrometer using electron ionization technique (Thermo Fisher Scientific). The m/z values of all the fragments with Sn were stated by the use of Sn = 119. The *S. aureus* (MTCC 9760) and *B. subtillis* (MTCC 1133), *E. coli* (MTCC 589) and *P. aeruginosa* (MTCC 9048), *A. niger* (MTCC 9933), and *S. canadensis* (MTCC 567) microbial Gram-positive, Gram-negative, and fungal traces, respectively were procured from IMTECH Chandigarh.

#### 3.1.1. Synthesis of Ligand (H_2_L)

**Scheme 1:**
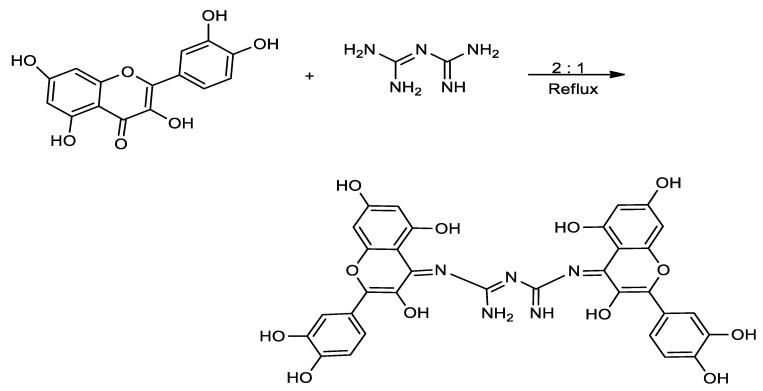
Synthesis of Schiff base.

A 250-mL, two-necked, round-bottomed flask and magnetic stirrer with hot plate were used to synthesize the compounds. Briefly, 2 mol quercetin. 2H_2_O was dissolved in 100 mL MeOH. The content was stirred for 20 min to yield a clear, dark, yellow solution. Next, 1 mol biguanide methanolic solution was dropwise added to the same flask until a yellow color was observed. Thereafter, the content was refluxed by non-stop stirring for approximately 3 h, and then stirring at room temperature for approximately 4 h. The content was maintained in a rotary vacuum evaporator until the complete evaporation of the solvent resulted in a yellow product. Finally, the obtained product was dried and subjected to spectroscopic and other studies. (Scheme 1): Yield: 75%, light yellow color; mp: 198–201 °C; m/z [M+] calculated for [C_32_H_23_N_5_O_12_]: 669.13; found: 669.57; Anal calc. for [C_32_H_23_N_5_O_12_]; C 57.40; H 3.46; N 10.46; found: C 57.84; H 3.96; N 10.40: FT-IR data (v, cm −1); 3373 v(OH) phenolic; 1600 v(C=N) aromatic; 1657 v(C=N) aliphatic; 1299 v(C-O-C); 1 HNMR (500 MHz; DMSO-d6, ppm): 12.15(5-OH), 9.3(3-OH), 8.31(7-OH), 7.6(2’-H), 7.5(6’-H), 6.7(5’-H), 6.0(8-H), 6.0(6-H), 2.6(NH): 13 CNMR (500 MHz; DMSO-d6; ppm): 164.3(C=N), 160.2(C-7), 157.0(C-5), 148.9(C-3’), 157.0(C-four’), 148.9(C-9), 145.1(C-2), 136.1(C-3), 132(C-1’), 121.5(C-5’), 119.1(C-2’), 115.3(C-6’), 100.2(C-6), 99.9(C-10), 94.1(C-8); UV-Vis; λ max (DMSO 10-3), 365 and 262 nm.

#### 3.1.2. Synthesis of Diorganotin (IV) Derivatives

**Scheme 2:**
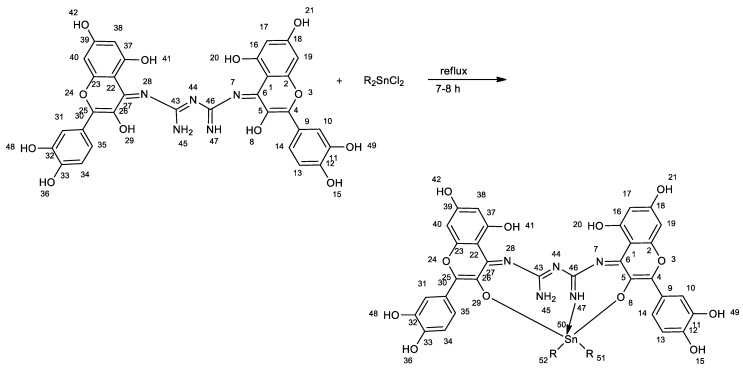
Synthesis of diorganotin (IV) complexes **1**–**4**. R = methyl (**1**), ethyl (**2**), butyl (**3**), phenyl (**4**).

The Schiff base ligand H_2_L (3 mmol) was dissolved in methanol solvent (25 mL) and stirred for approximately 20–30 min. Next, the above solution was refluxed with a methanolic solution of methyl (0.659 g, 3 mmol), ethyl (0.743 g, 3 mmol), butyl (0.911 g, 3 mmol), and phenyl (01.031 g, 3 mmol) derivatives of diorganotin (IV) dichloride. Then, a few drops of triethylamine were added to this solution and the content was refluxed for 7–8 h. After cooling, the obtained mixture was evaporated to half of its original volume under low pressure. Finally, the obtained orange-colored product was washed with methanol or diethyl ether and dried in vacuum (Scheme 2).

##### Me_2_SnL(**1**)

Yield: 72%, orange solid; mp: 175–178 °C; m/z [M+] calculated for [C_34_H_27_N_5_O_12_Sn]: 817.07; found: 819.05; Anal calc. for [C_34_H_27_N_5_O_12_Sn]; C 50.03; H 3.33; N 8.58; found: C 50.87; H 3.09; N 8.29: FT-IR data (v, cm-1); 3380 v(OH)phenolic; 1603 v(C=N) aromatic; 1630 v(C=N)aliphatic; 1298 v(C-O-C); 426 v(Sn-C); 509 v(Sn-O); 480 v(Sn-N); 1 HNMR (500 MHz; DMSO-d6, ppm): 12.18(5-OH), 8.17(7-OH), 7.1(2’-H), 7.0(6’-H), 6.5(5’-H), 6.3(8-H), 6.1(6-H), 2.4(NH), O.79(CH3-H): 13 CNMR (500 MHz; DMSO-d6; ppm): 162.3(C=N), 160.7(C-7), 157.0(C-5), 149.0(C-3’), 157.0(C-4’), 149.0(C-9), 145.4(C-2), 133.7(C-3), 132(C-1’), 121.4(C-5’), 119.2(C-2’), 115.4(C-6’), 100.8(C-6), 99.6(C-10), 94.3(C-8), 8.51(C-Me); 119 Sn nuclear magnetic resonance (400 MHz; DMSO; ppm): −140.43. UV-Vis; λ max (DMSO 10-3), 410 and 262 nm.

##### Et_2_SnL(**2**)

Yield: 76%, light orange solid; mp: 178–181 °C; m/z [M+] calculated for [C_36_H_31_N_5_O_12_Sn]: 845.10; found: 845.49; Anal calc. for [C_36_H_31_N_5_O_12_Sn]; C 51.21; H 3.70; N 8.29; found: C 51.73; H 4.30; N 8.59: FT-IR data (v, cm-1); 3382 v(OH)phenolic; 1605 v(C=N) aromatic; 1622 v(C=N)aliphatic; 1297 v(C-O-C); 428 v(Sn-C); 520 v(Sn-O); 467 v(Sn-N) 1 HNMR (500 MHz; DMSO-d6, ppm): 12.21(5-OH), 8.21(7-OH), 7.4(2’-H), 7.1(6’-H), 6.6(5’-H), 6.2(8-H), 6.2(6-H), 2.6(NH), O.88-0.90(Et-H): 13 CNMR (500 MHz; DMSO-d6; ppm): 162.5(C=N), 160.4(C-7), 157.2(C-5), 149.2(C-3’), 157.2(C-4’), 149.2(C-9), 145.8(C-2), 133.9(C-3), 133.1(C-1’), 121.8(C-5’), 119.8(C-2’), 115.6(C-6’), 100.6(C-6), 99.4(C-10), 94.7(C-8), 7.5(Me-C); 119 Sn NMR (400 MHz; DMSO; ppm): −190.68. UV-Vis; λ max (DMSO 10-3), 414 and 262 nm.

##### Bu_2_SnL(**3**)

Yield: 64%, dark orange solid; mp: 168–171 °C; m/z [M+] calculated for [C_40_H_39_N_5_O_12_Sn]: 901.16; found: 901.49; Anal calc. for [C_40_H_39_N_5_O_12_Sn]; C 53.35; H 4.37; N 7.78; found: C 54.73; H 4.48; N 7.59: FT-IR data (v, cm-1); 3384 v(OH)phenolic; 1607 v(C=N) aromatic; 1628 v(C=N)aliphatic; 1299 v(C-O-C); 482 v(Sn-C); 516 v(Sn-O); 460 v(Sn-N) 1 HNMR (500 MHz; DMSO-d6, ppm): 12.20(5-OH), 8.20(7-OH), 7.3(2’-H), 7.3(6’-H), 6.8(5’-H), 6.8(8-H), 6.6(6-H), 2.5(NH), O.9-2(Bu-H): 13 CNMR (500 MHz; DMSO-d6; ppm): 162.5(C=N), 160.4(C-7), 157.4(C-5), 149.0(C-3’), 157.4(C-4’), 149.0(C-9), 146.1(C-2), 134.1(C-3), 133.9(C-1’), 121.7(C-5’), 119.9(C-2’), 115.8(C-6’), 100.7(C-6), 99.9(C-10), 94.5(C-8), 33(Bu-C); 119 Sn NMR (400 MHz; DMSO; ppm): −213.47. UV-Vis; λ max (DMSO 10-3), 420 and 262 nm.

##### Ph_2_SnL(**4**)

Yield: 69%, orange solid; mp: 176–180 °C; m/z [M+] calculated for [C_44_H_31_N_5_O_12_Sn]: 941.10; found: 941.46; Anal calc. for [C_44_H_31_N_5_O_12_Sn]; C 56.19; H 3.32; N 7.45; found: C 56.73; H 3.68; N 7.65: FT-IR data (v, cm −1); 3394 v(OH)phenolic; 1612 v(C=N) aromatic; 1624 v(C=N)aliphatic; 1299 v(C-O-C); 463 v(Sn-C); 519 v(Sn-O); 473 v(Sn-N); 1 HNMR (500 MHz; DMSO-d6, ppm): 12.19(5-OH), 8.20(7-OH), 7.2(2’-H), 7.2(6’-H), 6.7(5’-H), 6.9(8-H), 6.8(6-H), 2.5(NH), 7.29–7.36(Ph-H): 13 CNMR (500 MHz; DMSO-d6; ppm): 163.5(C=N), 161.4(C-7), 157.8(C-5), 149.7(C-3’), 157.4(C-4’), 149.7(C-9), 146.2(C-2), 132.2(C-3), 133.7(C-1’), 121.9(C-5’), 119.6(C-2’), 115.6(C-6’), 100.5(C-6), 99.4(C-10), 94.9(C-8), 147.9–124.1(Ph-C); 119 Sn NMR (400 MHz; DMSO; ppm): −314. UV-Vis; λ max (DMSO 10-3), 430 and 262 nm.

### 3.2. Antimicrobial Activity

The in vitro antimicrobial screening of the newly designed ligand and its corresponding diorganotin (IV) derivatives were evaluated against different strains of microorganisms by determining the minimum inhibitory concentration (MIC). This activity has been evaluated using serial dilution and agar well diffusion technique [68]. The antibacterial activity was measured against Gram-positive species, such as *Staphylococcus aureus* (MTCC 9760), *Bacillus subtillis* (MTCC 1133), and Gram-negative strains, i.e., *Escherichia coli* (MTCC 589), *Pseudomonas aeruginosa* (MTCC 9048). The antifungal activity was carried out against fungal species, such as *Scopulariopsis canadensis* (MTCC 567) and *Aspergillus niger* (MTCC 9933). Ciprofloxacin and fluconazole were used as standard antimicrobials to compare the activities of tested compounds while 2% DMSO was used as a blank. The solutions were prepared by dissolving 5 mg of each compound in 5 mL of 2% DMSO as solvent. Then, 1 mL of the prepared solution was diluted with 9 mL of DMSO for the concentration of the solution to reach a value of up to 100 ppm [69,70].

### 3.3. Molecular Docking Studies

The crystal structures of dihydropteroate synthase (PDB ID: 1AD4), *E. coli* 24 kDa DNA gyrase domain (PDB ID: 1KZN), and UDP-*N-*acetylglucosamine pyrophosphorylase (6TN3) were obtained from the protein data bank and prepared for molecular docking using AutoDock Tool 1.5.6 [71]. Chain A of dihydropteroate synthase was determined as the ligand-binding domain for the docking study, and binding coordinates were assigned as center_x = 32.74; center_y = 7.91; center_z = 41.22. For *E. coli* 24 kDa DNA gyrase domain, the binding coordinates were assigned as center_x = 18.84; center_y = 24.05; center_z = 36.31. For UDP-*N-*acetylglucosamine pyrophosphorylase, the binding coordinates were assigned as center_x = 22.81; center_y = 60.59; center_z = −4.47. The search space was set up at 30 × 30 × 30 Å for all docking studies. Twenty possible binding conformations for each ligand were generated by AutoDock Vina suite software using genetic algorithm (GA-LS) searches [72,73]. Selected conformations of ligands and proteins were combined in Pymol software and analyzed in Discovery Studio Visualizer [74].

## 4. Structure–Activities Relationship

The back bone of the chemical structure of complexes is the presence of bi-flavonoid moiety, i.e., the presence of four benzene rings with two pyranone rings. The eight flavonoid hydroxyl groups present at 11, 12, 16, and 18th and 32, 33, 37, and 39th positions of each complex are responsible for anti-oxidative properties. Four >C=N bonds present at 6, 27, 43, and 46th positions provide excellent biocidal properties, whereas the double bonds present between the 4th and 5th positions and 25th and 26th positions of bi-flavonoid moiety are responsible for effective inhibitory activities [75]. Furthermore, complexation of Schiff base with organotin (IV) increases the biocidal potential of complex, which is possibly due to the increase in lipophilic character.

## 5. Conclusions

The current research involved the synthesis of novel, Schiff base ligand using quercetin and biguanide as initial materials. In the subsequent step, diorganotin (IV) complexes of the designed Schiff base ligand were synthesized. The spectroscopic records confirmed that the oxygen atom of the 3-OH group and imine nitrogen atoms are coordination sites for central tin atom, which result in the formation of penta-coordinated complexes. Antimicrobial screening displayed that the diorganotin (IV) complexes have greater inhibition potential than the Schiff base ligand, which may be due to the increase in lipophilicity and permeability through the cell membrane following the formation of complexes. The excessive lipid solubility of organotin compounds ensures better cell penetration and interaction with intercellular sites. Furthermore, the molecular docking proved that the complex formed in the case of **4** was the most effective agent as an antimicrobial along with the substantial interactions with DHPS (1AD4) receptor protein.

## Figures and Tables

**Table 1:** Antimicrobial screening data of the synthesized ligand and its diorganotin (IV) derivatives.

Compounds	Zone of Inhibition/mm (Concentration of Compounds 1–4) in µg/mL					
	Gram Positive		Gram Negative		Fungi	
	*S. aureus* 300 200 100	*B. subtilis* 300 200 100	*E. coli* 300 200 100	*Pseudomonas aeruginosa* 300 200 100	*S. canadesis* 300 200 100	*A. niger* 300 200 100
**H_2_L**	24 ± 0.4 19 ± 0.1 15 ± 0.5	23 ± 0.6 18 ± 0.9 14 ± 0.6	22 ± 0.5 20 ± 0.2 13 ± 0.7	22 ± 0.5 19 ± 0.4 13 ± 0.7	18 ± 0.7 17 ± 0.5 15 ± 0.6	19 ± 0.7 16 ± 0.4 14 ± 0.2
**1**	27 ± 0.5 22 ± 0.3 17 ± 0,5	25 ± 0.7 20 ± 0.8 17 ± 0.4	23 ± 0.6 21 ± 0.8 12 ± 0.7	24 ± 0.6 20 ± 0.8 13 ± 0.7	21 ± 0.8 19 ± 0.5 17 ± 0.7	22 ± 0.4 18 ± 0.3 16 ± 0.1
**2**	26 ± 0.9 21 ± 0.8 16 ± 0.7	26 ± 0.8 20 ± 0.3 17 ± 0.9	24 ± 0.9 21 ± 0.4 11 ± 0.7	24 ± 0.7 21 ± 0.8 13 ± 0.6	24 ± 0.4 22 ± 0.5 18 ± 0.5	18 ± 0.3 25 ± 0.3 18 ± 0.4
**3**	28 ± 0.9 27 ± 0.9 25 ± 0.8	28 ± 0.8 26 ± 0.6 21 ± 0.8	26 ± 0.7 24 ± 0.7 21 ± 0.6	26 ± 0.3 24 ± 0.6 18 ± 0.5	29 ± 0.6 26 ± 0.5 23 ± 0.5	28 ± 0.6 26 ± 0.5 23 ± 0.3
**4**	30 ± 0.9 27 ± 0.9 24 ± 0.7	30 ± 0.7 27 ± 0.4 23 ± 0.8	27 ± 0.7 24 ± 0.8 20 ± 0.2	27 ± 0.8 25 ± 0.8 21 ± 0.6	29 ± 0.6 26 ± 0.4 24 ± 0.4	30 ± 0.5 26 ± 0.8 24 ± 0.6
Ciprofloxacin (40 µg/mL)	28 mm	27 mm	27 mm	27 mm	____	______
Fluconazole (40 µg/mL)	______	_____	_____	______	28 mm	28 mm

**Table 2:** Molecular docking energies and interactions of the complexes with 1AD4, 1KZN, and 6TB3.

Receptors	1AD4		1KZN		6TN3	
Ligands	Binding Affinity (kcal/mol)	Interactions	Binding Affinity (kcal/mol)	Interactions	Binding Affinity (kcal/mol)	Interactions
H_2_L	−9.7	Conventional Hydrogen Bond (ASN A:103, MET A:128, and ARG A:204) Pi-Cation (ARG A:239) Pi-Pi T-Shaped (TTIS A:55 and PIIE A:172) Alkyl (MET A:128) Pi-Alkyl (ATA A:199)	−9.5	Conventional Hydrogen Bond (ASN A:46, ASP A:49, ASP A:73, and GLY A:77) Carbon Hydrogen Bond (HIS A:95 and GLY A:119) Pi-Anion (GLU A:50) Pi-Alkyl (ILE A:78 and ILE A:90)	−9.9	van der Waals (GLY B:137) Conventional Hydrogen Bond (THR B:140, ASN B:249, SER B:331, and LYS B:405) Pi-Cation (LYS B:383) Pi-Anion (GLU B:407) Pi-Sigma (GLN B:138) Amide-Pi Stacked (GLY B:136) Alkyl (ALA B:381) Pi-Alkyl (PRO B:246)
Me_2_SnL (**1**)	−8.4	Conventional Hydrogen Bond (VAL A:12, ARG A:66, and HIS A:241) Carbon Hydrogen Bond (LYS A:203) * Pi-Cation (ARG A:239)	−8.3	Conventional Hydrogen Bond (GLU A:42, ASN A:46, and ARG A:136) Pi-Cation (ARG A:76) Pi-Anion (ASP A:49 and GLU A:50) Pi-Alkyl (ILE A:78 and PRO A:79)	−9.2	Conventional Hydrogen Bond (GLY B:136, GLY B:137, THR B:140, GLN B:222, TYR B:330, LYS B:383, and GLU B:407) Pi-Cation (LYS B:383 and LYS B:437) Pi-Anion (ASP B:279) Pi-Sigma (ASN B:249) Pi-Sulfur (CYS B:277) Pi-Alkyl (VAL B:357)
Et_2_SnL (**2**)	−8.7	Conventional Hydrogen Bond (VAL A:12, ARG A:52, ARG A:66, ARG A:204, and ARG A:219) Pi-Cation (ARG A:52) Pi-Pi T-Shaped (PHE A:172) Pi-Alkyl (HIS A:55 and LYS A:203)	−9.0	Conventional Hydrogen Bond (ASP A:49, ILE A:90, VAL A:120, and SER A:121) Carbon Hydrogen Bond (GLY A:119) Pi-Donor Hydrogen Bond (ASN A:46) Pi-Sigma (ILE A:90) Pi-Alkyl (ALA A:96)	−9.2	Conventional Hydrogen Bond (GLY B:137, THR B:140, TYR B:330, LYS B:383, and GLU B:407) Carbon Hydrogen Bond (LYS B:383) Pi-Cation (LYS B:437) Pi-Anion (ASP B:279 and GLU B:407) Pi-Sigma (ASN B:249) Pi-Sulfur (CYS B:277) Pi-Alkyl (ARG B:141, VAL B:357, and LYS B:405)
Bu_2_SnL (**3**)	−8.4	Conventional Hydrogen Bond (GLY A:47, GLY A:48, ARG A:52, HIS A:55, ASP A:84, and ARG A:219) Pi-Alkyl (PRO A:216 and HIS A:241)	−8.5	Conventional Hydrogen Bond (ASN A:46, ASP A:49, and THR A:165) Carbon Hydrogen Bond (GLY A:119) Pi-Cation (ARG A:76) Pi-Anion (GLU A:50) Alkyl (ILE A:78 and PRO A:79) Pi-Alkyl (ILE A:78 and ILE A:90)	−9.1	Conventional Hydrogen Bond (MET B:134, THR B:140, GLN B:222, TYR B:330, LYS B:383, LYS B:405, and GLU B:407) Carbon Hydrogen Bond (GLY B:136 and LYS B:383) Pi-Cation (LYS B:383 and LYS B:437) Alkyl (PRO B:246) Pi-Alkyl (ARG B:141, CYS B:277, and VAL B:357)
Ph_2_SnL (**4**)	−8.9	Conventional Hydrogen Bond (GLY A:48, ARG A:52, HIS A:55, and ARG A:66) Pi-Alkyl (PRO A:216)	−8.3	Conventional Hydrogen Bond (ASN A:46) Pi-Cation (ARG A:76) Pi-Anion (GLU A:50) Pi-Alkyl (ILE A:78, PRO A:79, and ILE A:90)	−7.3	Conventional Hydrogen Bond (LYS B:383, ASN B:438, ALA B:439, and GLU B:444) Carbon Hydrogen Bond (ARG B:141) Pi-Pi T-shaped (TYR B:330) Pi-Alkyl (ARG B:141)

## Data Availability

The datasets generated during and/or analyzed during the current study are available from the corresponding author on reasonable request.

## Supplementary Materials

The following supporting information can be downloaded at: https://www.mdpi.com/article/10.3390/molecules27248874/s1, Figure S1: ^1^H NMR Spectra of H_2_L; Figure S2: IR Spectra of H_2_L; Figure S3: anti-bacterial activities of quercetin complexes; Figure S4: anti-bacterial activities of quercetin ligand (Schiff base).

## Sample Availability

Samples of the compounds are available with M.K. and Z.A.

## References

[1] Azeem M., Hanif M., Mahmood K., Ameer N., Chughtai F.R., Abid U. (2022). An insight into anticancer, antioxidant, antimicrobial, antidiabetic and anti-inflammatory effects of quercetin: A review. Polym. Bull..

[2] Al-Jumaili M.H.A., Hdeethi M.K.Y.A. (2021). Study of selected flavonoid structures and their potential activity as breast anticancer agents. Cancer Inform..

[3] Asgharian P., Tazehkand A.P., Soofiyani S.R., Hosseini K., Martorell M., Tarhriz V., Ahangari H. (2021). Quercetin impact in pancreatic cancer: An overview on its therapeutic effects. Oxidative Med. Cell. Longev..

[4] Bahare S., Machin L., Monzote L., Sharifi J., Ezzat M.S., Salem M.A., Rana M. (2010). Therapeutic Potential of Quercetin: New Insights and Perspectives for Human Health. ACS Omega.

[5] Daniel I.K., Özkömeç F.N., Çeşme M., Omowunmi A.S. (2022). Synthesis, biological and computational studies of flavonoid acetamide derivatives. RSC Adv..

[6] Balasubramanian R., Narayanan M., Kedalgovindaram L., Rama K.D. (2007). Cytotoxic activity of flavone glycoside from the stem of Indigofera aspalathoides Vahl. J. Nat. Med..

[7] Zhang H., Zhang M., Yu L., Zhao Y., He N., Yang X. (2012). Antitumor activities of quercetin and quercetin-5′, 8-disulfonate in human colon and breast cancer cell lines. Food Chem. Toxicol..

[8] Ahmedova A., Paradowska K., Wawer I. (2012). 1H, 13C MAS NMR and DFT GIAO study of quercetin and its complex with Al (III) in solid state. J. InorgBiochem..

[9] Carmen L., Arnoldi A. (2021). Food-derived antioxidants and COVID-19. J. Food Biochem..

[10] Tan J., Wang B., Zhu L. (2009). DNA binding, cytotoxicity, apoptotic inducing activity, and molecular modeling study of quercetin zinc (II) complex. Bioorg. Med. Chem..

[11] Bondžić A.M., Lazarević-Pašti T.D., Bondžić B.P., Čolović M.B., Jadranin M.B., Vasić V.M. (2013). Investigation of reaction between quercetin and Au (III) in acidic media: Mechanism and identification of reaction products. New J. Chem..

[12] Selvaraj S., Krishnaswamy S., Devashya V., Sethuraman S., Krishnan U.M. (2014). Flavonoid–metal ion complexes: A novel class of therapeutic agents. Med. Res. Rev..

[13] Santos N.E., Braga S.S. (2021). Redesigning Nature, Ruthenium Flavonoid Complexes with Antitumour, Antimicrobial and Cardioprotective Activities. Molecules.

[14] Dolatabadi J.E. (2011). Molecular aspects on the interaction of quercetin and its metal complexes with DNA, International journal of biological macromolecules. Int. J. Biol. Macromol..

[15] Xu D., Hu M.J., Wang Y.Q., Cui Y.L. (2019). Antioxidant activities of quercetin and its complexes for medicinal application. Molecules.

[16] Kasikci M.B., Bagdatlioglu N. (2016). Bioavailability of quercetin. Curr. Res. Nutr. Food Sci. J..

[17] Chen X., Yin O.Q., Zuo Z., Chow M.S. (2005). Pharmacokinetics and modeling of quercetin and metabolites. Pharm. Res..

[18] Massi A., Bortolini O., Ragno D., Bernardi T., Sacchetti G., Tacchini M., de Risi C. (2017). Research progress in the modification of quercetin leading to anticancer agents. Molecules.

[19] Park K.H., Choi J.M., Cho E., Jeong D., Shinde V.V., Kim H., Choi Y., Jung S. (2017). Enhancement of solubility and bioavailability of quercetin by inclusion complexation with the cavity of mono-6-deoxy-6-aminoethylamino-β-cyclodextrin. Bull. Korean Chem. Soc..

[20] Dian L., Yu E., Chen X., Wen X., Zhang Z., Qin L., Wang Q., Li G., Wu C. (2014). Enhancing oral bioavailability of quercetin using novel soluplus polymeric micelles. Nanoscale Res. Lett..

[21] Amanzadeh E., Esmaeili A., Abadi R.E., Kazemipour N., Pahlevanneshan Z., Beheshti S. (2019). Quercetin conjugated with superparamagnetic iron oxide nanoparticles improves learning and memory better than free quercetin via interacting with proteins involved in LTP. Sci. Rep..

[22] Sun L., Lu B., Liu Y., Wang Q., Li G., Zhao L., Zhao C. (2021). Synthesis, characterization and antioxidant activity of quercetin derivatives. Synth. Commun..

[23] Moodi Z., Bagherzade G., Peters J. (2021). Quercetin as a precursor for the synthesis of novel nanoscale Cu (II) complex as a catalyst for alcohol oxidation with high antibacterial activity. Bioinorg. Chem. Appl..

[24] Wang Q., Huang Y., Zhang J.S., Yang X.B. (2014). Synthesis, characterization, DNA interaction, and antitumor activities of La (III) complex with Schiff base ligand derived from kaempferol and diethylenetriamine. Bioinorg. Chem. Appl..

[25] Folkman J. (1971). Tumor angiogenesis: Therapeutic implications. N. Engl. J. Med..

[26] Ambika S., Kumar Y.M., Arunachalam S., Gowdhami B., Sundaram K.K., Solomon R.V., Venuvanalingam P., Akbarsha M.A., Sundararaman M. (2019). Biomolecular interaction, anti-cancer and anti-angiogenic properties of cobalt (III) Schiff base complexes. Sci. Rep..

[27] Duaa G., Zahraa R., Emad Y. (2018). A review of organotin compounds:chemistry and applications. Arch. Org. Inorg. Chem. Sci..

[28] Adeyemi J.O., Onwudiwe D.C. (2018). Organotin(IV) Dithiocarbamate Complexes: Chemistry and Biological Activity. Molecules.

[29] Kumar M., Abbas Z., Tuli H.S., Rani A. (2020). Organotin Complexes with Promising Therapeutic Potential. Curr. Pharmacol. Rep..

[30] Valentina U., Alexandra-Cristina M. (2017). Flavonoid Complexes as Promising Anticancer Metallodrugs. Flavonoids—From Biosynthesis to Human Health.

[31] (2016). Flavonoid-Based Organometallics with Different Metal Centers—Investigations of the Effects on Reactivity and Cytotoxicity. Eur. J. Inorg. Chem..

[32] Lionta E., Spyrou G., Vassilatis D.K., Cournia Z. (2014). Structure-based virtual screening for drug discovery: Principles, applications and recent advances. Curr. Top. Med. Chem..

[33] Paul A., Anbu S., Sharma G., Kuznetsov M.L., Koch B., da Silva M.F., Pombeiro A.J. (2015). Synthesis, DNA binding, cellular DNA lesion and cytotoxicity of a series of new benzimidazole-based Schiff base copper (II) complexes. Dalton Trans..

[34] Devi J., Yadav M., Kumar A. (2018). Synthesis, characterization, biological activity, and QSAR studies of transition metal complexes derived from piperonylamine Schiff bases. Chem. Pap..

[35] Singh H.L., Singh J.B., Bhanuka S. (2016). Synthesis, spectroscopic characterization, biological screening, and theoretical studies of organotin (IV) complexes of semicarbazone and thiosemicarbazones derived from (2-hydroxyphenyl)(pyrrolidin-1-yl) methanone. Res. Chem. Intermed..

[36] Nakamoto K. (2009). Infrared and Raman Spectra of Inorganic and Coordination Compounds, Part B: Applications in Coordination, Organometallic, and Bioinorganic Chemistry.

[37] Nath M., Vats M., Roy P. (2015). Mode of action of tin-based anti-proliferative agents: Biological studies of organotin (IV) derivatives of fatty acids. J. Photochem. Photobiol. B Biol..

[38] De Souza R.F., de Giovani W.F. (2005). Synthesis, spectral and electrochemical properties of Al (III) and Zn (II) complexes with flavonoids. Spectrochim. Acta Part A Mol. Biomol. Spectrosc..

[39] Tan Y.X., Zhang Z.J., Liu Y., Yu J.X., Zhu X.M., Kuang D.Z., Jiang W.J. (2017). Synthesis, crystal structure and biological activity of the Schiff base organotin (IV) complexes based on salicylaldehyde-o-aminophenol. J. Mol. Struct..

[40] Abbas Z., Tuli H.S., Varol M., Sharma S., Sharma H.K., Aggarwal P., Kumar M. (2021). Organotin (IV) complexes derived from Schiff base 1, 3-bis [(1E)-1-(2-hydroxyphenyl) ethylidene] thiourea: Synthesis, spectral investigation and biological study to molecular docking. J. Iran. Chem. Soc..

[41] Singh K., Dharampal, Parkash V. (2008). Synthesis, spectroscopic studies, and in vitro antifungal activity of organosilicon (IV) and organotin (IV) complexes of 4-amino-5-mercapto-3-methyl-S-triazole Schiff bases. Phosphorus Sulfur Silicon Relat. Elem..

[42] Wagler J., Böhme U., Brendler E., Thomas B., Goutal S., Mayr H., Kempf B., Remennikov G.Y., Roewer G. (2005). Switching between penta-and hexacoordination with salen-silicon-complexes. Inorg. Chim. Acta.

[43] Sirajuddin M., Ali S., Shah F.A., Ahmad M., Tahir M.N. (2014). Potential bioactive Vanillin–Schiff base di-and tri-organotin (IV) complexes of 4-((3, 5-dimethylphenylimino) methyl)-2-methoxyphenol: Synthesis, characterization and biological screenings. J. Iran. Chem. Soc..

[44] Devi J., Yadav J., Kumar D., Jindal D.K., Basu B. (2020). Synthesis, spectral analysis and in vitro cytotoxicity of diorganotin (IV) complexes derived from indole-3-butyric hydrazide. Appl. Organomet. Chem..

[45] Kang J., Liu Y., Xie M.X., Li S., Jiang M., Wang Y.D. (2004). Interactions of human serum albumin with chlorogenic acid and ferulic acid. Biochim. Biophys. Acta (BBA)-Gen. Subj..

[46] Bukhari S.B., Memon S., Mahroof-Tahir M., Bhanger M.I. (2009). Synthesis, characterization and antioxidant activity copper-quercetin complex. Spectrochim. Acta Part A Mol. Biomol. Spectrosc..

[47] Dehghan G., Khoshkam Z. (2012). Tin (II)–quercetin complex: Synthesis, spectral characterisation and antioxidant activity. Food Chem..

[48] Ahmadi S.M., Dehghan G., Hosseinpourfeizi M.A., Dolatabadi J.E., Kashanian S. (2011). Preparation, characterization, and DNA binding studies of water-soluble quercetin–molybdenum (VI) complex. DNA Cell Biol..

[49] Cornard J.P., Merlin J.C. (2002). Spectroscopic and structural study of complexes of quercetin with Al (III). J. Inorg. Biochem..

[50] Kumar M., Aggarwal P., Varol M., Sharma S., Rani A., Abbas Z., Prakash V., Tuli H.S. (2022). Synthesis, spectral investigation, biological potential and molecular docking study of novel Schiff base and its transition metal complexes. Anti-Infect. Agents.

[51] Aggarwal P., Tuli H.S., Kumar M. (2022). Novel Cyclic Schiff Base and Its Transition Metal Complexes: Synthesis, Spectral and Biological Investigations. Iran. J. Chem. Chem. Eng..

[52] Anjaneyulu Y., Rao R.P. (1986). Preparation, characterization and antimicrobial activity studies on some ternary complexes of Cu (II) with acetylacetone and various salicylic acids. Synth. React. Inorg. Met. Org. Chem..

[53] Chohan Z.H., Scozzafava A., EnzyInhib C.T.S.J. (2003). Zinc complexes of benzothiazole-derived Schiff bases with antibacterial activity. J. Enzym. Inhib. Med. Chem..

[54] Chilwal A., Malhotra P., Narula A.K. (2014). Synthesis, characterization, thermal and antibacterial studies of organotin (IV) complexes of indole-3-butyric acid and indole-3-propionic acid. Phosphorus Sulfur Silicon Relat. Elem..

[55] Shujah S., Zia-ur-Rehman M., Ali N., Khalid S., Tahir M.N. (2011). New dimeric and supramolecular organotin (IV) complexes with a tridentate schiff base as potential biocidal agents. J. Organomet. Chem..

[56] Gilad Y., Senderowitz H. (2014). Docking studies on DNA intercalators. J. Chem. Inf. Model..

[57] Minovski N., Perdih A., Novic M., Solmajer T. (2013). Cluster-based molecular docking study for in silico identification of novel 6-fluoroquinolones as potential inhibitors against Mycobacterium tuberculosis. J. Comput. Chem..

[58] Baginski M., Fogolari F., Briggs J.M. (1997). Electrostatic and non-electrostatic contributions to the binding free energies of anthracycline antibiotics to DNA. J. Mol. Biol..

[59] Rehn C., Pindur U. (1996). Molecular modeling of intercalation complexes of antitumor active 9-aminoacridine and a [d,e]-anellated isoquinoline derivative with base paired deoxytetranucleotides. Mon. Chem. Chem. Mon..

[60] Waring M.J., Bailly C. (1994). The purine 2-amino group as a critical recognition element for binding of small molecules to DNA. Gene.

[61] Boer D.R., Canals A., Coll M. (2009). DNA-binding drugs caught in action: The latest 3D pictures of drug-DNA complexes. Dalton Trans..

[62] Satari M.H., Apriyanti E., Dharsono H.D., Nurdin D., Gartika M., Kurnia D. (2021). Effectiveness of bioactive compound as antibacterial and anti-quorum sensing agent from myrmecodia pendans: An in silico study. Molecules.

[63] Patil T.D., Amrutkar S.V. (2022). Novel Benzotriazole Acetamide Derivatives as Benzo-Fused Five-Membered Nitrogen-Containing Heterocycles-In silico Screening, Molecular Docking, and Synthesis. Lett. Drug Des. Discov..

[64] Ahsan M.J., Yusuf M., Salahuddin Bakht M.A., Taleuzzaman M., Vashishtha B., Thiriveedhi A. (2022). Green Synthesis, Biological Evaluation, and Molecular Docking of 4’-(Substituted Phenyl) Spiro [Indoline-3, 3′-[1, 2, 4] Triazolidine]-2, 5′-Diones. Polycycl. Aromat. Compd..

[65] Latgé J.P., Chamilos G. (2019). Aspergillus fumigatus and Aspergillosis in 2019. Clin. Microbiol. Rev..

[66] Raimi O.G., Hurtado-Guerrero R., Borodkin V., Ferenbach A., Urbaniak M.D., Ferguson M.A., van Aalten D.M. (2020). A mechanism-inspired UDP-N-acetylglucosamine pyrophosphorylase inhibitor. RSC Chem. Biol..

[67] Armarego W.L., Chai C.L.L. (2009). Purification of Laboratory Chemicals, Butterworth-Heinemann.

[68] Jorgensen J.H., Turnidge J.D., Murray P.R., Baron E.J., Pfaller M.A., Tenover F.C., Yolken R.H. (1999). Manual of Clinical Microbiology.

[69] Devi J., Yadav M., Kumar D., Naik L.S., Jindal D.K. (2019). Some divalent metal (II) complexes of salicylaldehyde-derived Schiff bases: Synthesis, spectroscopic characterization, antimicrobial and in vitro anticancer studies. Appl. Organomet. Chem..

[70] Kumar M., Pallvi T.H.S., Khare R. (2019). Synthesis, Characterization and Biological Studies of Novel Schiff Base viz. Bis-1, 1′-(pyridine-2, 6-diyldieth-1-yl-1-ylidene) biguanidine and Their Transition Metal Complexes. Asian J. Chem..

[71] Morris G.M., Huey R., Lindstrom W., Sanner M.F., Belew R.K., Goodsell D.S., Olson A.J. (2009). AutoDock4 and AutoDockTools4: Automated docking with selective receptor flexibility. J. Comput. Chem..

[72] Mukherjee S., Mitra I., Saha R., Dodda S.R., Linert W., Moi S.C. (2015). In vitro model reaction of sulfur containing bio-relevant ligands with Pt(ii) complex: Kinetics, mechanism, bioactivity and computational studies. RSC. Adv..

[73] Eberhardt J., Santos-Martins D., Tillack A.F., Forli S. (2021). New docking methods, expanded force field, and python bindings. J. Chem. Inf. Model..

[74] Macindoe G., Mavridis L., Venkatraman V., Devignes M.D., Ritchie D.W. (2010). HexServer: An FFT-based protein docking server powered by graphics processors. Nucleic Acids Res..

[75] Das A., Baidya R., Chakraborty T., Samanta A.K., Roy S. (2021). Pharmacological basis and new insights of taxifolin: A comprehensive review. Biomed. Pharmacother..

