# Supplementary material for: Synthesis, Structural Investigations, and In Vitro/In Silico Bioactivities of Flavonoid Substituted Biguanide: A Novel Schiff Base and Its Diorganotin (IV) Complexes
Source: Molecules. 2022 Dec 14;27(24):8874. doi: 10.3390/molecules27248874 (PMC9783859; doi:10.3390/molecules27248874)
Supplement: Supplementary file 1 [file molecules-27-08874-s001.zip › molecules-2061893-supplementary.pdf]

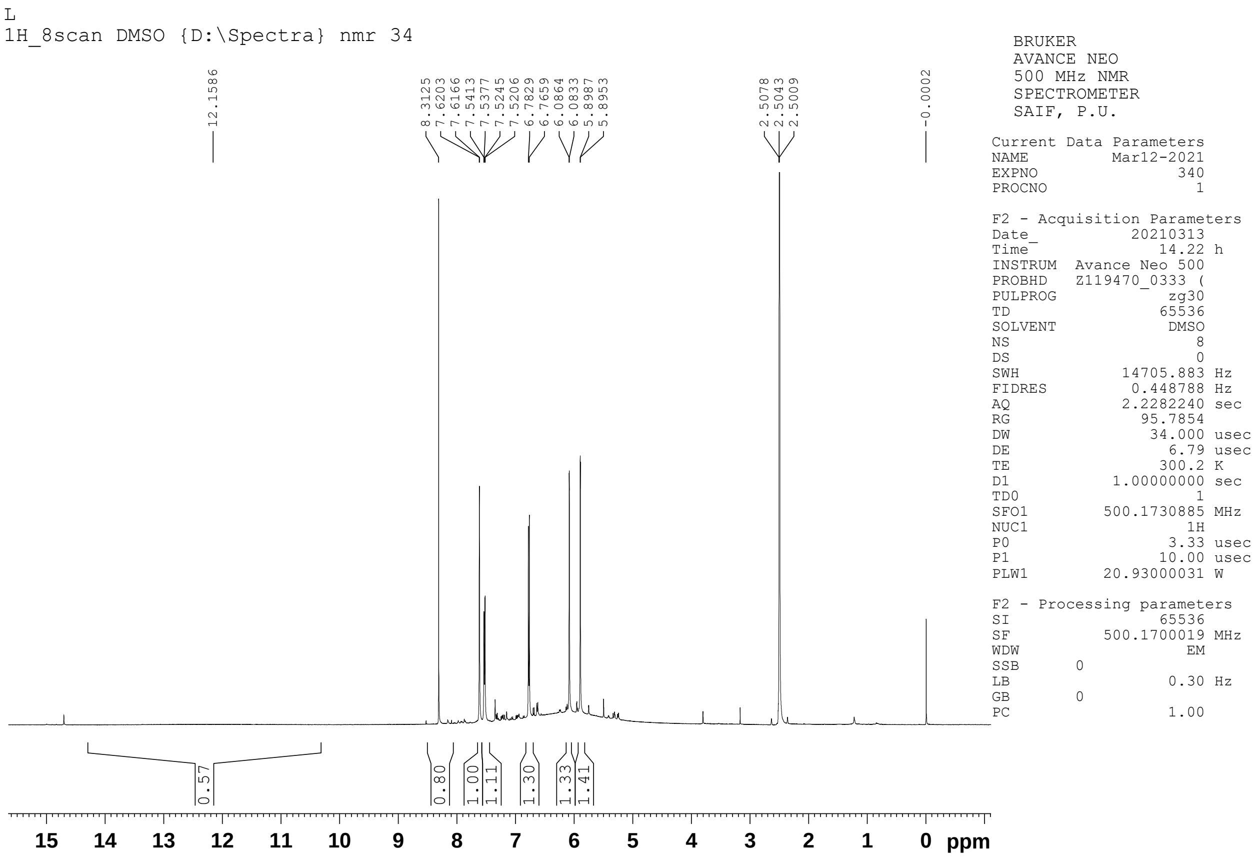

Figure S1: ^{1}H NMR Spectra of H_2L.

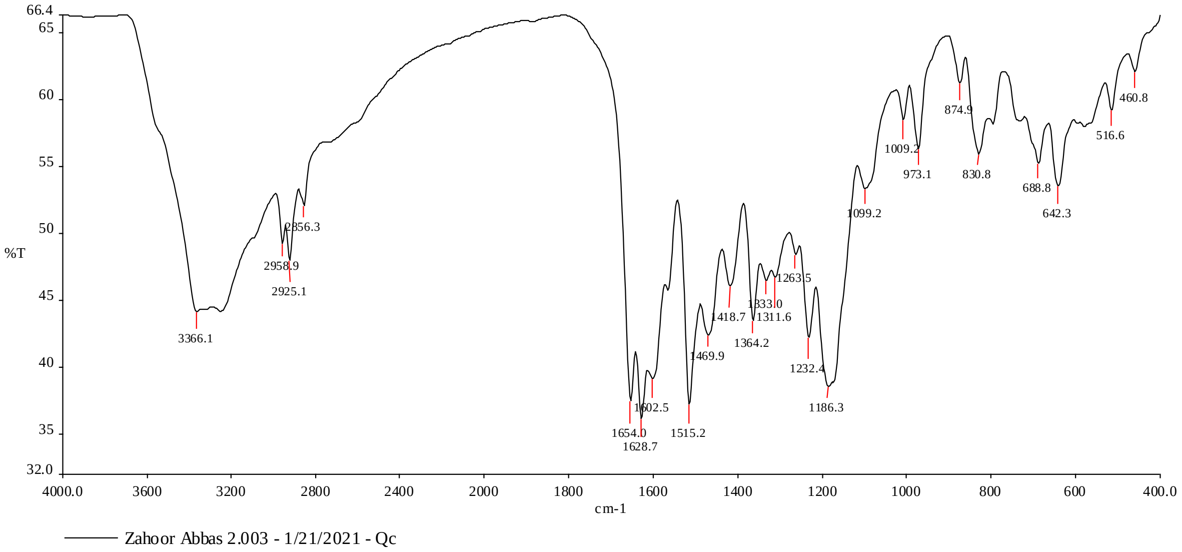

Figure S2: IR Spectra of H_2L.

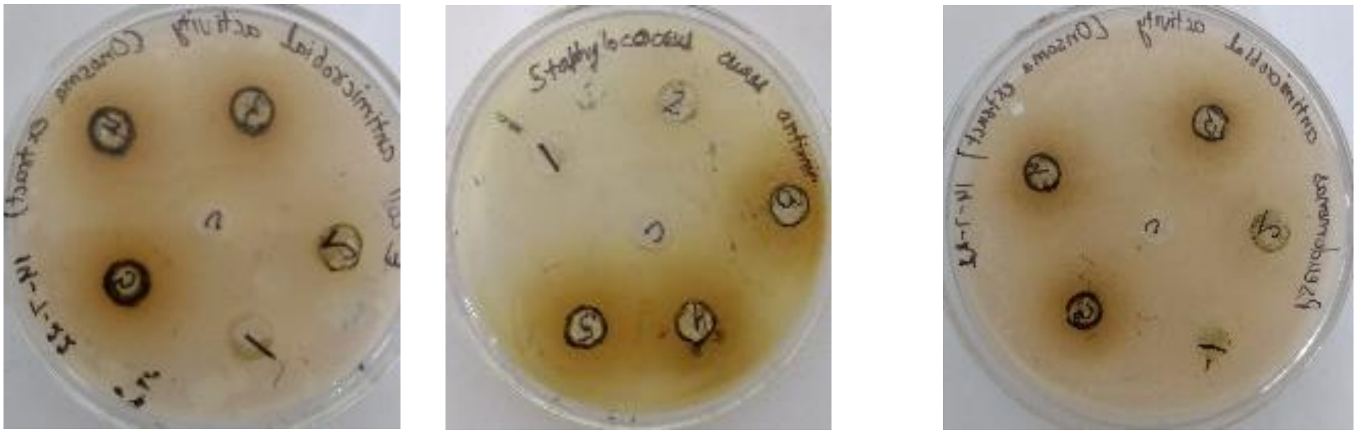

Figure S3: anti-bacterial activities of quercetin complexes.

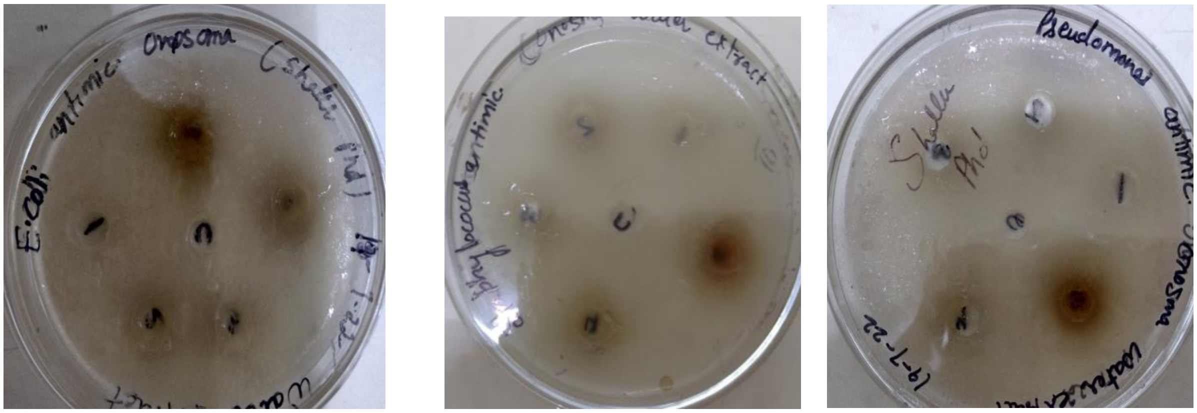

Figure S4: anti-bacterial activities of quercetin ligand (Schiff base).
